# Associations Between Mental-Health Factors, Quality of Life, and Suicidal Ideation in Korean Elderly

**DOI:** 10.3390/healthcare14183109

**Published:** 2026-09-20

**Authors:** Seung-Hyun Cho

**Affiliations:** Department of Occupational Therapy, College of Welfare and Education, Tongmyong University, Busan 48520, Republic of Korea; kaic21@gmail.com

**Keywords:** elderly, mental health, quality of life, suicidal ideation, depressive symptoms, Korea Community Health Survey

## Abstract

**Background/Objectives**: This study examined associations of mental health and health-related quality of life (HRQOL) with suicidal ideation among older Korean adults, and whether an HRQOL difference persisted after adjustment for depressive symptoms. **Methods**: The 2022 Korea Community Health Survey included 79,441 adults aged ≥65 years. The outcome was a past-year report of thoughts of wanting to die, which may include passive death wishes. Complex-samples logistic models used categorical PHQ-8, self-rated health, and stress (*n* = 78,838). An exploratory design-based linear analysis estimated adjusted EQ-5D differences (*n* = 79,033). **Results**: The weighted outcome prevalence was 9.9% (95% CI 9.6–10.2). Standardized prevalences across the PHQ-8 categories were 6.5%, 13.6%, 18.7%, and 23.9%; the highest versus lowest category difference was 17.4 percentage points (95% CI 14.3–20.5). Stress and EQ-5D anxiety/depression were positively associated with the outcome, whereas happiness was inversely associated (aOR 0.770, 95% CI 0.751–0.790). The adjusted EQ-5D mean difference for respondents with versus without the outcome was −0.0531 (95% CI −0.0589 to −0.0473). The primary mental-health associations persisted with expanded covariates; some physical-domain estimates depended on specification or variance handling. **Conclusions**: Depressive symptoms, stress, anxiety/depression, and happiness were associated with the outcome, and an HRQOL difference persisted after depressive-symptom adjustment. These mutually adjusted, cross-sectional findings concern a single item that may include passive death wishes and do not establish causal effects or a clinical diagnosis.

## 1. Introduction

Suicide mortality is a public health concern in Korea as its population ages [1,2]. Population studies have documented long-term increases in suicide mortality in Korea and Japan [3], while recent Korean research has reported associations of suicide-related thoughts in later life with depressive symptoms and socioeconomic circumstances [2,4]. Examining how mental health and physical functioning accompany these thoughts can provide context for community assessment of older adults.

Suicide-related thoughts range from passive death wishes to active thoughts involving a method, intent, or plan. DSM-5-TR includes recurrent thoughts of death and suicidal ideation within criterion A9 for a major depressive episode [5], whereas the Columbia–Suicide Severity Rating Scale distinguishes a wish to be dead from active suicidal thoughts [6]. KCHS studies have reported suicidal ideation among older adults [7], including use of the term for past-year thoughts of wanting to die [8]. This study retains that operational terminology for comparability. The item may include passive death wishes and does not establish active ideation, intent, planning, a psychiatric diagnosis, or a comprehensive assessment of suicidality.

Older adults reporting suicidal ideation also report poorer physical and mental HRQOL [9,10,11]. Recent Korean studies have examined depressive symptoms, health-risk behaviors, chronic disease, and social isolation [2,4,12,13]. Longitudinal research has considered social disconnection and suicide mortality in Japan [14], and suicidal ideation, mobility limitations, and social participation in Europe [15]. A 2024 systematic review identified well-being, life satisfaction, positive relationships, and social support as potentially protective correlates [16]. This literature supports considering mental health alongside social circumstances and functioning, although measures of distress, subjective well-being, and HRQOL partly overlap.

Earlier national-survey studies in older Korean adults examined HRQOL alongside reported depressive diagnoses, or identified correlates using a binary measure of depressive experience [9,17]. Estimates across depressive-symptom levels and of the accompanying HRQOL difference can help characterize the health concerns associated with thoughts of wanting to die. The primary analysis therefore reports standardized prevalences across PHQ-8 severity categories; an exploratory secondary analysis quantifies the EQ-5D mean difference after adjustment for the PHQ-8 score. Both analyses require attention to measurement overlap: PHQ-9 item 9 addresses thoughts of death or self-harm [18], so the primary analysis uses the PHQ-8, which omits that item [19]. Sequential and sensitivity models assess how the findings depend on adjustment and specification.

Using data from 79,441 KCHS participants aged ≥65 years, this study addressed two questions. The primary analysis examined how depressive symptoms, self-rated health, happiness, perceived stress, and the five EQ-5D dimensions were associated with the operational suicidal-ideation outcome after mutual adjustment. An exploratory secondary analysis examined whether the adjusted EQ-5D index differed between respondents with and without the outcome after accounting for depressive symptoms and sociodemographic and clinical characteristics. Standardized prevalences were used to express the primary associations on a probability scale, and additional models assessed their sensitivity to adjustment, functional form, and the EQ-5D ceiling.

## 2. Materials and Methods

### 2.1. Study Design and Data Source

The 2022 Korea Community Health Survey (KCHS) raw dataset was analyzed in this cross-sectional secondary study [20]. The KDCA administers this government-approved survey each year under the Regional Public Health Act; the target population comprises adults aged 19 years or older who live in areas served by public health centers throughout Korea. A stratified cluster design is used for sampling, and trained interviewers collect responses in one-to-one, face-to-face interviews. The 2022 dataset contained 231,785 adults aged 19 years or older.

### 2.2. Study Population

**Figure 1:**
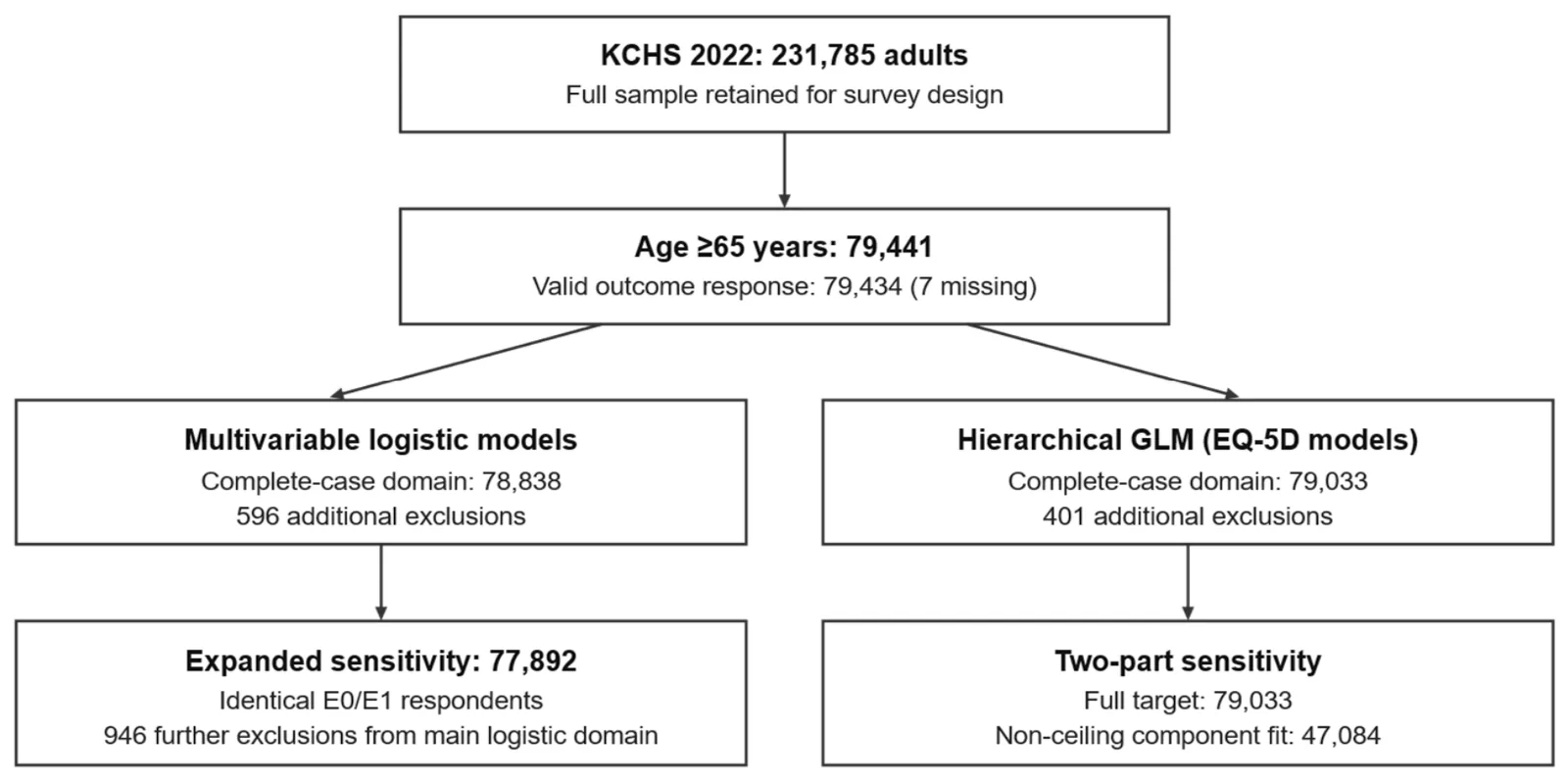
Flow of participant selection and table-specific analysis domains.

The study population comprised 79,441 respondents aged ≥65 years. Younger respondents were retained in the survey-design object, with older adults specified as a subpopulation. Seven participants had missing outcome responses, leaving 79,434. Complete-case domains contained 78,838 participants for the multivariable logistic models and 79,033 for the hierarchical GLM with 596 and 401 additional exclusions, respectively. Both domains required valid PHQ-8 and PHQ-9 totals so that PHQ sensitivity analyses compared the same respondents. Among outcome-valid respondents, PHQ-8, PHQ-9, and happiness had 325, 328, and 246 missing observations, respectively; these counts can overlap. Table S1 summarizes missingness and included–excluded comparisons, and Figure 1 shows the analysis domains.

### 2.3. Variable Definitions and Measures

#### 2.3.1. Suicidal Ideation (Dependent Variable)

The dependent variable was constructed from the KCHS item, “During the past year, have you ever thought about wanting to die?” A yes response was coded 1 and no 0. Previous KCHS studies have used suicidal ideation for this outcome [7,8]; this study uses the term in that operational sense. The item may include passive death wishes and does not assess method, intent, planning, behavior, severity, or frequency [5,6]. It is neither a DSM-5-TR diagnosis nor a comprehensive assessment of suicidality.

#### 2.3.2. Depressive Symptoms (PHQ-8/PHQ-9)

Each Patient Health Questionnaire item is scored from 0 (not at all) to 3 (nearly every day) for the preceding 2 weeks [21]. The PHQ-9 covers the nine depressive-symptom domains, but a clinical diagnosis also requires assessment of symptom combinations, impairment, duration, exclusions, and differential diagnoses [5]. PHQ-9 item 9 addresses thoughts of death or self-harm [18]. The primary measure was the PHQ-8 sum of the remaining items (0–24) [19], grouped as 0–4, 5–9, 10–14, and 15–24. The two upper standard bands were combined because the five-band IBM SPSS Statistics (version 29.0.2; IBM Corp., Armonk, NY, USA) fit was numerically unstable. A linear-programming diagnostic found no separation, and a stable five-band R (version 4.6.0; R Foundation for Statistical Computing, Vienna, Austria) fit was retained as a sensitivity analysis (Table S2). A missing component made the relevant total missing.

#### 2.3.3. Health-Related Quality of Life (EQ-5D)

Health status was recorded with the EuroQol-5 Dimension (EQ-5D) across mobility, self-care, usual activities, pain/discomfort, and anxiety/depression; each dimension was rated as “no problems,” “some problems,” or “severe problems.” For logistic regression, “no problems” served as the reference and indicator variables represented the other two categories. Overall quality-of-life impairment and the sensitivity analyses were evaluated with the EQ-5D index based on Korean population tariffs [22]; an index value nearer 1 indicates a health state nearer perfect health.

#### 2.3.4. Self-Rated Health

The item “How would you rate your health in general?” provided the measure of self-rated health. Its 5-point response scale extended from “very good” to “very poor.” Reverse coding produced scores for which higher values represented better self-rated health (range, 1–5).

#### 2.3.5. Happiness

In the KCHS, happiness is recorded with a single item on a 10-point scale; response options extend from 1 (very unhappy) to 10 (very happy).

#### 2.3.6. Perceived Stress

One KCHS item measured perceived stress. It asked, “How much stress do you usually feel in your daily life?” with four response levels from “very much” to “hardly any.” Responses were reverse-coded so that a higher score denoted greater perceived stress on the resulting 4-level scale (range, 1–4).

#### 2.3.7. Sociodemographic Characteristics and Covariates

The common demographic and clinical covariates were sex, age, education, spousal cohabitation, receipt of basic livelihood security benefits, economic activity, and physician-diagnosed hypertension and diabetes. Selection was based on prior Korean older-adult studies and availability in the 2022 KCHS [2,4,9,12,13,23]. An expanded sensitivity model additionally included one-person household status, marital status (cohabiting spouse, widowed, or other), subjective cognitive decline, past-year alcohol use (none, occasional, or frequent), current smoking, log-transformed monthly household income, and urban/rural residence. EQ-5D physical domains provided indicators of functional limitation. Psychiatric diagnoses, objective cognitive testing, detailed social support/contact, bereavement timing, substance-use disorders, and a comprehensive multimorbidity measure were unavailable.

### 2.4. Statistical Analysis

All analyses incorporated the survey stratum (kstrata), primary sampling unit (SPOT_NO), and individual weight (wt_p). The full sample contained 5631 strata and 22,589 nested PSUs; 1080 strata contained one sampled PSU. Variances were estimated by with-replacement Taylor linearization. The primary convention followed IBM SPSS Statistics by assigning zero variance contribution to single-PSU strata, matched in R with survey.lonely.psu = “remove”. Respondents and strata were retained in point estimation. A sensitivity analysis used conservative grand-mean centering for single-PSU strata (Table S2). Full-design and logistic regression domain degrees of freedom were 16,958 and 14,181, respectively; these were distinguished from regression residual degrees of freedom.

Participant characteristics were summarized as survey-weighted means or category percentages with standard errors. Continuous variables were compared using design-based linear Wald tests and categorical variables using Rao–Scott F tests. R scripts used Taylor linearization, and representative batched descriptive estimates were checked against direct survey-package calculations. Missing values were not imputed. Comparisons of included and excluded respondents described possible selection differences rather than correcting selection bias.

To address the first question, the primary logistic model included PHQ-8 categories (reference 0–4), five self-rated-health categories (reference very good), four stress categories (reference hardly any), continuous happiness, and all EQ-5D dimension indicators (reference no problems). Age and happiness were centered at 75 and 7 for numerical stability. The adjustment sequence began with demographic and clinical covariates (M1), then added PHQ-8 (M2), self-rated health, happiness, and stress (M3), the four physical EQ-5D dimensions (M4), and anxiety/depression (M5). The last dimension was entered separately because its content overlaps depressive symptoms. All five models used the same complete-case logistic domain. The sequence describes changes with adjustment and does not assume a causal or temporal order.

Additional models assessed sensitivity to functional form and covariate selection. Using the same complete-case domains, these analyses examined continuous PHQ-8/self-rated-health/stress terms, PHQ-9 replacement, the EQ-5D index instead of its dimensions, five PHQ-8 bands, and quadratic terms. Selected estimates are reported in Table S2, with complete coefficients for the index-replacement model in Table S3. The targeted usual-activities sequence began with demographic/clinical covariates and usual activities, then added categorical mental-health measures, the other physical EQ-5D dimensions, and anxiety/depression. The expanded comparison used the same 77,892 respondents in E0 and E1: E0 replaced spousal cohabitation with marital-status indicators, and E1 additionally included one-person household status, subjective cognitive decline, alcohol use, smoking, income, and rural residence (Table S2).

Hierarchical general linear models addressed the second question on the common GLM analysis domain. These exploratory models estimated the adjusted EQ-5D mean difference by outcome status. M1 included outcome status, PHQ-8, sex, and age; M2 added socioeconomic covariates; and M3 added hypertension and diabetes. Design-based standard errors accommodated heteroscedasticity. The estimand was a mean difference, for which health-utility research supports linear analysis despite a non-normal outcome distribution [24]. Diagnostics included observed and fitted ranges, the ceiling proportion, and weighted residual root mean square error. In a two-part sensitivity analysis, a logistic model for full health was fitted in the full domain and a linear model for the index below 1 in the non-ceiling domain. For each assigned outcome status, the models generated p_i, the predicted probability of full health, and m_i, the predicted non-ceiling index, for every respondent in the full domain. The combined prediction, p_i + (1 − p_i)m_i, was then averaged with survey weights. Joint component uncertainty was not estimated, so combined contrasts were reported as descriptive point estimates without a confidence interval.

**Figure 2:**
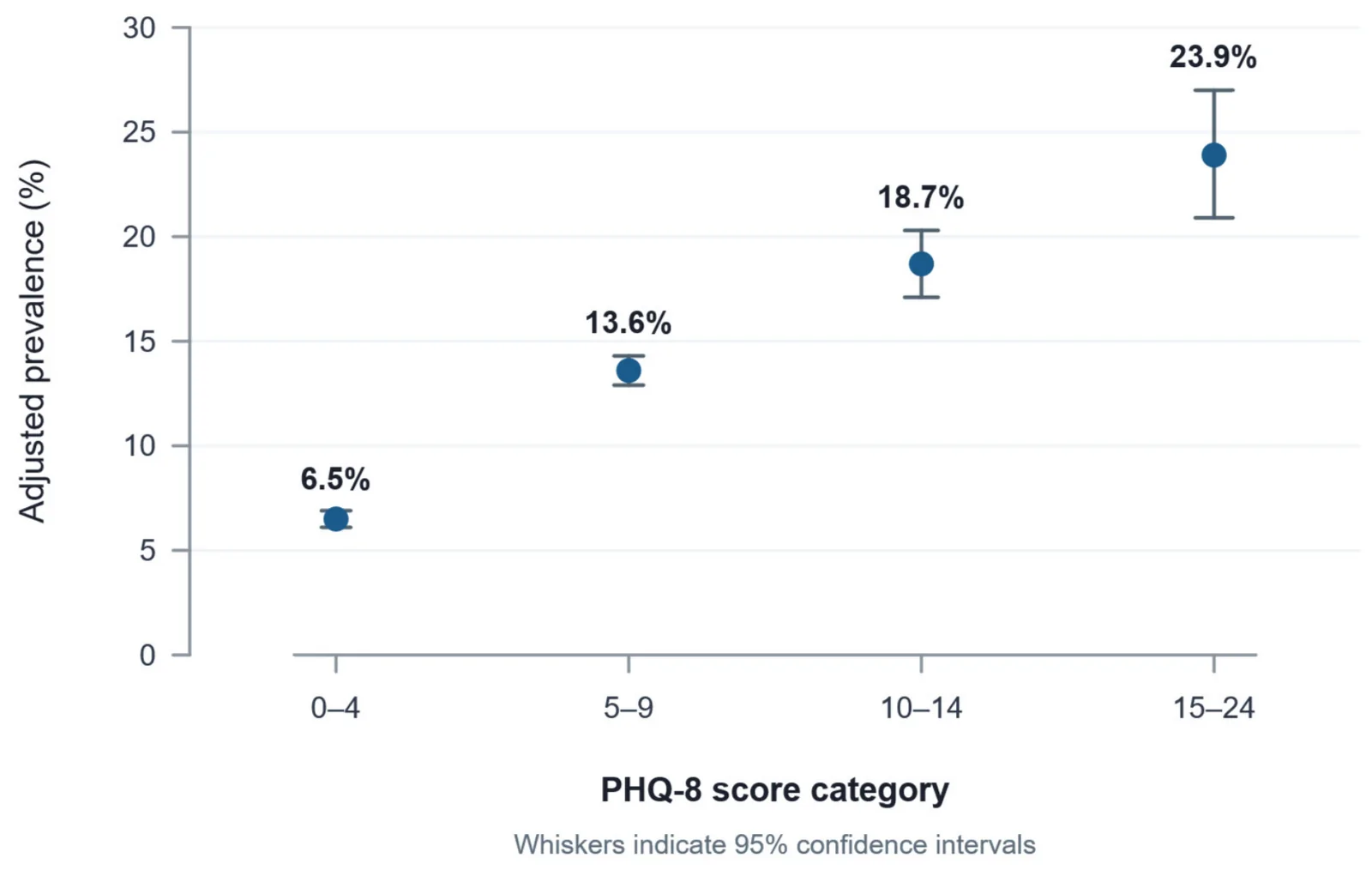
The standardized predicted prevalence of reporting past-year thoughts of wanting to die across PHQ-8 categories. The points and whiskers show survey-weighted predictions and 95% CIs from the fully adjusted logistic model (M5; *n* = 78,838). Each respondent was assigned the indicated PHQ-8 category while other covariates retained their observed values. These model-based standardized associations differ from observed category-specific prevalences and do not represent intervention effects. The outcome may include passive death wishes.

Standardized prevalences were calculated from the primary logistic model by assigning every respondent a given PHQ-8, stress, or EQ-5D anxiety/depression category, retaining their other observed covariates, and averaging predicted probabilities with survey weights [25]. Thus, comparisons used the same observed covariate distribution. Logit-scale confidence intervals obtained by Taylor linearization were transformed back to probabilities; prevalence differences used joint prediction covariance and a t critical value with 14,181 degrees of freedom. Figure 2 displays the PHQ-8 estimates. These quantities describe standardized associations, not intervention effects. IBM SPSS Statistics produced the primary regression estimates; R with the survey package (version 4.5) produced predictions and specified sensitivities. Coefficients, standard errors, covariance matrices, and inferential results were checked under matched conventions. Benjamini–Hochberg adjustment covered all 30 non-intercept M5 coefficients [26]. Descriptive, block, and sensitivity analyses were not separate confirmatory FDR families. All tests were two-sided, with a primary *q* threshold of 0.05.

### 2.5. Ethical Considerations

This study was conducted in accordance with the ethical principles of the Declaration of Helsinki and was approved for exemption from review by the Institutional Review Board of Tongmyong University (approval no. 202409-HR-001). The publicly available KCHS data used in this analysis were fully de-identified and did not permit identification of individual participants. Participants provided informed consent during the original survey.

## 3. Results

### 3.1. General Characteristics of the Study Population

Among 79,434 older respondents with a valid outcome response, 7913 reported having thought about wanting to die during the past year. Weighted prevalence was 9.9% (95% CI 9.6–10.2). This operational suicidal-ideation outcome may include passive death wishes. Table 1 describes the two response groups using variable-specific valid denominators.

Respondents reporting the outcome had higher mean age (75.29 vs. 73.71 years) and PHQ-8 score (7.23 vs. 2.26), lower happiness (4.93 vs. 6.90), and a lower mean EQ-5D index (0.725 vs. 0.892) than those not reporting it. Very poor or poor self-rated health was reported by 64.3% versus 29.0%, and much or very much stress by 46.8% versus 11.9%, respectively (all design-adjusted *p* < 0.001). Adjusted associations are reported below.

### 3.2. Factors Associated with Suicidal Ideation: Fully Adjusted Complex-Samples Logistic Regression

Higher PHQ-8 categories were associated with higher odds of reporting thoughts of wanting to die in the fully adjusted model (Table 2). Relative to scores of 0–4, aORs were 2.61 for 5–9, 4.14 for 10–14, and 6.05 for 15–24. Each contrast met the FDR threshold (all *q* < 0.001).

Stress and EQ-5D anxiety/depression were also positively associated with the outcome. Compared with hardly any stress, aORs were 1.47 for a little, 2.49 for much, and 2.65 for very much stress. For EQ-5D anxiety/depression, aORs were 2.25 for some and 4.06 for severe problems relative to no problems. Happiness was inversely associated in the linear specification (aOR 0.770 per point, 95% CI 0.751–0.790). All of these coefficients had *q* < 0.001.

The standardized predicted prevalences across the PHQ-8 categories were 6.5%, 13.6%, 18.7%, and 23.9% (Figure 2). The highest versus lowest category difference was 17.4 percentage points (95% CI 14.3–20.5). Standardized prevalence also increased across the stress categories (7.0%, 9.2%, 13.3%, and 13.8%) and the EQ-5D anxiety/depression categories (7.5%, 13.5%, and 20.0%; Table S2). These estimates use the same observed covariate distribution and concern the past-year thoughts-of-wanting-to-die item, which may include passive death wishes.

Self-rated health did not show a monotonic pattern across categories. Its overall adjusted Wald F was 8.12 (*p* < 0.001), but only good versus very good health met the category-level FDR threshold (aOR 0.668, *q* = 0.036). Among physical EQ-5D dimensions, some mobility problems met the threshold (aOR 1.156, *q* = 0.036), whereas severe mobility problems and both self-care categories did not. Severe usual-activity problems had an inverse coefficient (aOR 0.695, *q* = 0.020), examined below. Both pain/discomfort categories met the primary FDR threshold (*q* = 0.042 and 0.046), but not under conservative single-PSU centering (*q* = 0.057 and 0.063; Table S2). All 30 *q* values, including nonsignificant results, are shown in Table 2.

PHQ-8 coefficients were smaller after self-rated health, happiness, and stress were added and decreased further when EQ-5D anxiety/depression entered the model (Table 3). The depressive-symptom, stress, anxiety/depression, and happiness associations nevertheless retained their direction in the expanded-covariate comparison (Table S2). For happiness, the aOR was 0.771 in E0 and 0.778 in E1 (both *p* < 0.001), compared with 0.770 in the primary model.

In the targeted usual-activities sequence, the aOR for severe problems was 6.141 after demographic/clinical adjustment, 1.080 after adding mental-health measures, 0.776 after the other physical EQ-5D dimensions, and 0.695 after anxiety/depression (Table S2). By contrast, observed weighted outcome prevalences rose from 6.1% with no usual-activity problems to 20.5% with some and 32.1% with severe problems. The inverse fully adjusted coefficient therefore depended on extensive mutual adjustment and did not indicate a protective association.

The conventional five-band R sensitivity produced finite, stable estimates and a similar overall PHQ-8 pattern. In the continuous sensitivities, the per-point aOR was 1.175 for PHQ-8 and 1.203 for PHQ-9. The PHQ-8 quadratic term was significant. Within the categorical primary specification, the happiness quadratic term was nominally significant (*p* = 0.023), whereas the age quadratic term was not (Table S2). The linear happiness coefficient therefore summarizes an average conditional association across the observed scores; it should not be interpreted as a constant gradient at every score.

### 3.3. Difference in Health-Related Quality of Life by Suicidal-Ideation Status (Exploratory Secondary Analysis)

The adjusted mean EQ-5D index was lower among respondents reporting thoughts of wanting to die, even after accounting for PHQ-8 and sociodemographic and clinical characteristics. On the common domain of 79,033 respondents, the mean difference was −0.0585 in Model 1, −0.0534 in Model 2, and −0.0531 in Model 3 (all *p* < 0.001; Table 4). The final 95% CI was −0.0589 to −0.0473. The PHQ-9 replacement produced a difference of −0.0432 (95% CI −0.0490 to −0.0374; Table S2).

The EQ-5D index ranged from −0.171 to 1, with 43.1% of weighted observations at 1. GLM fitted values ranged from 0.298 to 1.041; 3538 predictions (4.5%) exceeded 1 and none fell below −0.171. The weighted residual root mean square error was 0.132. These diagnostics limit individual-level prediction, although the primary estimand was the adjusted mean difference.

Standardizing both two-part components over all 79,033 respondents gave combined means of 0.8806 without the outcome and 0.8301 with it, a descriptive difference of −0.0506 (Table S2). Predictions were combined for each respondent before averaging. The direction and magnitude were similar to the primary GLM estimate. Table S3 reports the component coefficients; no joint confidence interval or significance test was assigned to the combined contrast.

No interaction between outcome status and PHQ-8 was detected in the fully adjusted model (B = −0.0003, 95% CI −0.0018 to 0.0012, *p* = 0.703); PHQ-9 replacement was also nonsignificant (B = 0.0008, *p* = 0.258). These results provide no statistical evidence that the adjusted EQ-5D difference varies with PHQ score on the additive index scale. They do not establish equivalence.

## 4. Discussion

This national sample of community-dwelling older adults provided two findings. Depressive symptoms, stress, and EQ-5D anxiety/depression were positively associated with the KCHS suicidal-ideation item, while happiness was inversely associated in the linear specification. The outcome concerns past-year thoughts of wanting to die and may include passive death wishes. Figure 2 expresses the magnitude of the depressive-symptom association: standardized prevalence increased from 6.5% in the lowest PHQ-8 category to 23.9% in the highest. In the exploratory secondary analysis, a lower EQ-5D index was associated with reporting the outcome after adjustment for PHQ-8 and other covariates, with a final mean difference of −0.0531.

The overall weighted prevalence of 9.9% was similar to estimates from previous Korean older-adult surveys [9,23]. Such comparisons depend on item wording and recall period, particularly whether passive death wishes are included. The present prevalence and regression estimates concern the operational KCHS item; they should not be interpreted as estimates of active suicidal intent or a psychiatric diagnosis.

Standardized prevalences express the PHQ-8 association on a probability scale that is easier to interpret than an odds ratio alone. When predictions used the same observed covariate distribution, the highest and lowest categories differed by 17.4 percentage points. This contrast does not estimate the effect of changing a person’s PHQ-8 score. The stress and happiness findings are consistent with Korean research on depressive symptoms and life satisfaction [2], health-risk behaviors, depression, and anxiety [12], and happiness in relation to suicidal ideation across age groups [27]. Happiness was inversely associated in the expanded model, although the quadratic sensitivity cautions against assuming a constant gradient across its full range. PHQ-8 omits the death/self-harm item [19]; the larger per-point PHQ-9 estimate does not by itself establish why the estimates differ.

An HRQOL difference was evident after depressive-symptom adjustment, answering the second research question. Earlier studies have also reported poorer HRQOL among older adults with suicidal ideation [9,10,11]. The PHQ-8-adjusted mean contrast complements comparisons by reported depressive diagnosis [9] by quantifying the accompanying HRQOL difference while accounting for measured symptom severity. The mean difference changed little across the three adjustment stages, and the two-part standardized point estimate had a similar direction and magnitude. A linear model provides a directly interpretable mean contrast [24], although predictions outside the index range limit its use for individual utility prediction. Because the index itself includes anxiety/depression, the residual difference cannot be attributed solely to physical health or interpreted as an independent causal burden after adjustment for depressive symptoms.

Each coefficient in the mutually adjusted model describes an association conditional on the other included measures. PHQ-8 estimates decreased when well-being measures and EQ-5D anxiety/depression were added, which is compatible with shared content across measures. Logistic coefficients can also change because of noncollapsibility; their attenuation does not quantify confounding, mediation, or overadjustment [28]. The nonmonotonic self-rated-health estimates likewise do not support a uniform gradient across all categories. The block and functional-form analyses show how interpretation depends on the model specification.

Usual activities provide a concrete example of this dependence. Observed weighted outcome prevalence increased with greater usual-activity problems, whereas the aOR for severe problems changed from 6.141 with demographic/clinical adjustment to 0.695 in the fully adjusted model. Overlapping measures or suppression may contribute to this pattern [29], but the analysis does not identify a causal mechanism. Functional limitation should not be interpreted as protective against suicide-related thoughts. The pain/discomfort findings also require qualification because their FDR status changed under conservative single-PSU variance handling.

The expanded model included one-person household status, marital status, subjective cognitive decline, alcohol use, smoking, income, and rural residence. The main mental-health associations persisted, but these variables are incomplete proxies: living alone does not directly measure social isolation, current widowhood does not identify bereavement timing, and subjective cognitive decline is not objective cognitive impairment. Recent Korean and international studies support examining social connections, chronic disease, mobility, participation, and psychosocial well-being alongside depressive symptoms [4,13,14,15,16]. The findings provide descriptive context for community assessment of older adults. The models were not developed or validated as screening tools, and the benefit of combining assessment approaches was not tested.

### 4.1. Limitations

First, the cross-sectional design does not establish temporal ordering, causality, mediation, or directionality. The PHQ refers to the preceding 2 weeks, whereas the outcome refers to the preceding year, further limiting temporal interpretation. Second, the single outcome item cannot separate passive death wishes from active ideation and contains no information on severity, frequency, intent, plan, or behavior. Third, PHQ-8 measures symptoms rather than a diagnosed disorder, and the mental-health, well-being, and EQ-5D measures overlap. Mutual adjustment may produce highly conditional associations; noncollapsibility also complicates comparison of logistic coefficients. Fourth, the EQ-5D index was bounded with a marked ceiling, and some GLM fitted values exceeded 1. The two-part sensitivity supported the direction but did not provide a joint confidence interval.

Fifth, residual confounding is possible despite expanded adjustment. The 2022 file lacked psychiatric history, objective cognitive testing, detailed social support/contact, bereavement timing, substance-use disorders, and a comprehensive physical-disease profile. Sixth, complete-case analysis may introduce selection bias. Excluded respondents were older and reported the outcome more often (Table S1), so small exclusion proportions do not establish that missingness was ignorable. Seventh, institutionalized older adults were not sampled. Eighth, pain/discomfort inference depended on single-PSU variance handling; findings from multiple comparisons in a single survey year require cautious interpretation and replication.

### 4.2. Future Research Directions

Longitudinal studies should establish temporal ordering among depressive symptoms, social circumstances, functional decline, HRQOL, and suicide-related thoughts. Future surveys should use validated multidimensional instruments that distinguish passive death wishes from active ideation, method, intent, plan, and behavior, together with diagnostic assessment of depressive and other psychiatric disorders. Replication is needed in institutionalized and clinically characterized older adults and with measured social support, bereavement, objective cognition, alcohol/substance disorders, and multimorbidity. Prediction studies should separately evaluate discrimination, calibration, clinical utility, and implementation before any screening pathway is proposed.

## 5. Conclusions

Among community-dwelling older Korean adults, depressive symptoms, perceived stress, and EQ-5D anxiety/depression were positively associated with self-reported suicidal ideation that may include passive death wishes. Happiness was inversely associated in the linear specification. Standardized prevalence increased across PHQ-8 categories from 6.5% to 23.9%, and an adjusted EQ-5D mean difference of −0.0531 persisted after accounting for depressive symptoms and other covariates. These estimates add detail to population evidence on the depressive-symptom gradient and the HRQOL difference accompanying thoughts of wanting to die. The single-item, cross-sectional data do not establish causal effects or active suicidal intent.

## Figures and Tables

**Table 1:** Weighted characteristics of adults aged ≥65 years by suicidal ideation, 2022 Korea Community Health Survey (eligible *n* = 79,441; suicidal-ideation-valid *n* = 79,434).

Characteristic	No Suicidal Ideation (*n* = 71,521)	Suicidal Ideation (*n* = 7913)	F	*p*
Sex: Men, % (SE)	46.6 (0.19)	35.6 (0.70)	206.59	<0.001
Sex: Women, % (SE)	53.4 (0.19)	64.4 (0.70)		
Age, years, mean (SE)	73.71 (0.036)	75.29 (0.109)	190.95	<0.001
Education: Elementary school or below, % (SE)	39.8 (0.27)	56.3 (0.78)	197.10	<0.001
Education: Middle/high school, % (SE)	45.6 (0.27)	36.3 (0.74)		
Education: College or higher, % (SE)	14.5 (0.22)	7.4 (0.47)		
Living with spouse: Yes, % (SE)	66.6 (0.26)	48.9 (0.80)	498.21	<0.001
Basic livelihood security recipient: Yes, % (SE)	7.2 (0.16)	17.4 (0.61)	504.04	<0.001
Economically active: Yes, % (SE)	33.8 (0.24)	20.6 (0.60)	330.71	<0.001
Diagnosed hypertension: Yes, % (SE)	53.7 (0.25)	58.9 (0.76)	42.08	<0.001
Diagnosed diabetes: Yes, % (SE)	23.9 (0.22)	29.8 (0.70)	71.52	<0.001
PHQ-8 score, mean (SE)	2.26 (0.02)	7.23 (0.08)	3654.62	<0.001
PHQ-8 category: 0–4, % (SE)	83.6 (0.19)	34.2 (0.71)	2536.44	<0.001
PHQ-8 category: 5–9, % (SE)	13.8 (0.18)	38.7 (0.73)		
PHQ-8 category: 10–14, % (SE)	2.1 (0.07)	16.6 (0.57)		
PHQ-8 category: 15–24, % (SE)	0.5 (0.04)	10.5 (0.49)		
EQ-5D index, mean (SE)	0.892 (0.0007)	0.725 (0.0033)	2426.21	<0.001
Self-rated health: Very poor, % (SE)	5.2 (0.10)	22.9 (0.61)	747.06	<0.001
Self-rated health: Poor, % (SE)	23.8 (0.22)	41.4 (0.74)		
Self-rated health: Fair, % (SE)	42.2 (0.26)	26.9 (0.68)		
Self-rated health: Good, % (SE)	25.8 (0.23)	7.9 (0.43)		
Self-rated health: Very good, % (SE)	2.9 (0.09)	1.0 (0.15)		
Happiness score, mean (SE)	6.90 (0.01)	4.93 (0.03)	3700.87	<0.001
Perceived stress: Hardly any, % (SE)	40.1 (0.25)	14.3 (0.51)	1394.63	<0.001
Perceived stress: A little, % (SE)	48.0 (0.26)	39.0 (0.72)		
Perceived stress: Much, % (SE)	10.7 (0.16)	36.8 (0.72)		
Perceived stress: Very much, % (SE)	1.2 (0.06)	10.0 (0.50)		
Mobility: No problems, % (SE)	70.3 (0.23)	38.7 (0.77)	1054.78	<0.001
Mobility: Some problems, % (SE)	28.7 (0.23)	56.7 (0.77)		
Mobility: Severe problems, % (SE)	1.0 (0.04)	4.7 (0.29)		
Self-care: No problems, % (SE)	90.2 (0.14)	69.6 (0.69)	969.08	<0.001
Self-care: Some problems, % (SE)	8.7 (0.13)	25.6 (0.65)		
Self-care: Severe problems, % (SE)	1.1 (0.05)	4.8 (0.27)		
Usual activities: No problems, % (SE)	78.7 (0.20)	46.9 (0.77)	1268.83	<0.001
Usual activities: Some problems, % (SE)	19.7 (0.19)	46.6 (0.76)		
Usual activities: Severe problems, % (SE)	1.5 (0.06)	6.5 (0.32)		
Pain/discomfort: No problems, % (SE)	53.3 (0.26)	22.6 (0.66)	1319.25	<0.001
Pain/discomfort: Some problems, % (SE)	42.5 (0.25)	57.3 (0.74)		
Pain/discomfort: Severe problems, % (SE)	4.2 (0.10)	20.1 (0.59)		
Anxiety/depression: No problems, % (SE)	86.4 (0.18)	39.9 (0.76)	3136.55	<0.001
Anxiety/depression: Some problems, % (SE)	13.2 (0.17)	50.8 (0.76)		
Anxiety/depression: Severe problems, % (SE)	0.5 (0.04)	9.3 (0.45)		

The values are survey-weighted means or percentages with standard errors (SE); group *n* values are unweighted. Variable-specific valid denominators were used. The categorical comparisons use Rao–Scott F tests and the continuous comparisons use design-based linear Wald tests. These descriptive *p* values are exploratory. The outcome is the past-year thoughts-of-wanting-to-die item and may include passive death wishes.

**Table 2:** Fully adjusted complex-samples logistic regression for factors associated with suicidal ideation among adults aged ≥65 years (*n* = 78,838).

Parameter	aOR (95% CI)	*p*	*q*
**Mental health and well-being**			
PHQ-8: 5–9	2.610 (2.382–2.860)	<0.001	<0.001
PHQ-8: 10–14	4.141 (3.572–4.799)	<0.001	<0.001
PHQ-8: 15–24	6.052 (4.887–7.494)	<0.001	<0.001
Self-rated health: very poor	1.092 (0.774–1.541)	0.617	0.662
Self-rated health: poor	0.870 (0.624–1.212)	0.410	0.492
Self-rated health: fair	0.810 (0.583–1.127)	0.211	0.264
Self-rated health: good	0.668 (0.476–0.936)	0.019	0.036
Happiness, per point (centered at 7)	0.770 (0.751–0.790)	<0.001	<0.001
Stress: a little	1.471 (1.330–1.626)	<0.001	<0.001
Stress: much	2.488 (2.205–2.806)	<0.001	<0.001
Stress: very much	2.651 (2.153–3.263)	<0.001	<0.001
**EQ-5D dimensions**			
Mobility: some problems	1.156 (1.025–1.303)	0.018	0.036
Mobility: severe problems	1.097 (0.808–1.490)	0.553	0.638
Self-care: some problems	1.007 (0.895–1.133)	0.905	0.936
Self-care: severe problems	1.087 (0.810–1.457)	0.579	0.643
Usual activities: some problems	1.085 (0.961–1.225)	0.187	0.255
Usual activities: severe problems	0.695 (0.528–0.914)	0.009	0.020
Pain/discomfort: some problems	1.130 (1.016–1.257)	0.024	0.042
Pain/discomfort: severe problems	1.211 (1.021–1.436)	0.028	0.046
Anxiety/depression: some problems	2.254 (2.050–2.478)	<0.001	<0.001
Anxiety/depression: severe problems	4.061 (3.185–5.177)	<0.001	<0.001
**Sociodemographic and clinical covariates**			
Women (ref: men)	0.939 (0.864–1.021)	0.143	0.205
Age, per year (centered at 75)	0.991 (0.985–0.998)	0.007	0.016
Middle/high school	0.871 (0.799–0.950)	0.002	0.004
College or higher	0.753 (0.636–0.890)	<0.001	0.002
Living with spouse	0.733 (0.672–0.798)	<0.001	<0.001
Basic livelihood security recipient	1.106 (0.984–1.244)	0.091	0.137
Economically active	0.942 (0.861–1.032)	0.198	0.258
Diagnosed hypertension	0.998 (0.922–1.080)	0.960	0.960
Diagnosed diabetes	1.099 (1.007–1.198)	0.033	0.053

All 30 non-intercept coefficients form one Benjamini–Hochberg family. Reference categories: PHQ-8 0–4; very good self-rated health; hardly any stress; no problems in each EQ-5D dimension; men; and elementary education or below. Binary indicators compare yes with no. Age and happiness were centered at 75 and 7. The complete-case domain contains 78,838 respondents. The overall adjusted Wald F tests for PHQ-8, self-rated health, and stress were 208.56, 8.12, and 80.58 (all *p* < 0.001). Table S2 reports the targeted usual-activities sequence and the pain/discomfort sensitivity to single-PSU variance handling.

**Table 3:** Stability of selected associations across theory-informed sequential logistic models (*n* = 78,838).

Parameter	M1	M2	M3	M4	M5
Women (ref.: men)	1.08 (1.01–1.17)	0.92 (0.85–0.99)	0.99 (0.91–1.07)	0.95 (0.87–1.03)	0.94 (0.86–1.02)
Age, per year (centered at 75)	1.00 (1.00–1.01)	0.99 (0.98–1.00)	1.00 (0.99–1.00)	0.99 (0.98–1.00)	0.99 (0.99–1.00)
PHQ-8: 5–9	NA	6.09 (5.64–6.58)	3.20 (2.93–3.49)	3.04 (2.78–3.32)	2.61 (2.38–2.86)
PHQ-8: 10–14	NA	16.20 (14.39–18.23)	5.75 (5.01–6.61)	5.31 (4.62–6.12)	4.14 (3.57–4.80)
PHQ-8: 15–24	NA	41.59 (34.65–49.92)	9.59 (7.86–11.71)	8.86 (7.22–10.86)	6.05 (4.89–7.49)
Stress: a little	NA	NA	1.64 (1.49–1.81)	1.62 (1.47–1.79)	1.47 (1.33–1.63)
Stress: much	NA	NA	3.15 (2.80–3.55)	3.09 (2.74–3.47)	2.49 (2.21–2.81)
Stress: very much	NA	NA	3.60 (2.95–4.41)	3.51 (2.86–4.30)	2.65 (2.15–3.26)
Usual activities: some problems	NA	NA	NA	1.15 (1.02–1.30)	1.09 (0.96–1.23)
Usual activities: severe problems	NA	NA	NA	0.78 (0.59–1.02)	0.69 (0.53–0.91)
Anxiety/depression: some problems	NA	NA	NA	NA	2.25 (2.05–2.48)
Anxiety/depression: severe problems	NA	NA	NA	NA	4.06 (3.19–5.18)
Nagelkerke pseudo-R^2^	0.060	0.254	0.334	0.339	0.354

The cells are aOR (95% CI); NA indicates a variable not included in the model. M1: demographic/clinical covariates. M2: +PHQ-8 categories. M3: +self-rated health, happiness, and stress. M4: +the four physical EQ-5D dimensions. M5: +anxiety/depression. All the models use identical respondents; the sequence is not a mediation analysis. Table S2 reports the targeted usual-activities sequence, which begins before usual activities enter M4.

**Table 4:** Hierarchical design-based GLM for the adjusted difference in EQ-5D index associated with the operational suicidal-ideation outcome among adults aged ≥65 years (*n* = 79,033).

Parameter	Model 1	Model 2	Model 3
Suicidal-ideation item: yes vs. no	−0.0585 *** (−0.0643 to −0.0526)	−0.0534 *** (−0.0592 to −0.0476)	−0.0531 *** (−0.0589 to −0.0473)
PHQ-8, per point	−0.0191 *** (−0.0197 to −0.0185)	−0.0182 *** (−0.0188 to −0.0176)	−0.0181 *** (−0.0186 to −0.0175)
R^2^	0.344	0.360	0.362

The cells are B (95% CI); *** *p* < 0.001 for the individual exploratory GLM coefficient, without the Table 2 FDR adjustment. Model 1: suicidal-ideation item, PHQ-8, sex, and age. Model 2: +education, spousal cohabitation, basic livelihood security, and economic activity. Model 3: +hypertension and diabetes. All the models use the same 79,033 respondents. The outcome-status coefficient is the adjusted EQ-5D mean difference for yes versus no on the thoughts-of-wanting-to-die item. Table S2 summarizes PHQ replacement, interaction, and combined two-part estimates; Table S3 reports all the coefficients of the two-part components.

## Data Availability

The de-identified 2022 KCHS data were obtained from the Korea Disease Control and Prevention Agency (KDCA) under its data-use conditions. The author cannot redistribute these individual-level data. Researchers seeking access must apply directly through the KCHS data-access portal (https://chs.kdca.go.kr/chs/rdr/rdrInfoProcessMain.do (accessed on 24 September 2024)) and obtain approval from KDCA.

## Supplementary Materials

The following supporting information can be downloaded at https://www.mdpi.com/article/10.3390/healthcare14183109/s1: Table S1 summarizes missingness and analysis-domain inclusion. Selected sensitivity estimates and additional standardized prevalences are reported in Table S2, and Table S3 provides all coefficient estimates for the EQ-5D index-replacement model and the two components of the two-part model.

## References

[1] OECD (2024). Suicide Rates (Indicator).

[2] Park E.-Y., Choi S.-Y., Kim J.-H. (2026). Determinants of suicidal ideation in older adults: Evidence from a national panel study in South Korea. Front. Public Health.

[3] Jeon S.Y., Reither E.N., Masters R.K. (2016). A population-based analysis of increasing rates of suicide mortality in Japan and South Korea, 1985–2010. BMC Public Health.

[4] Kim E., Yi J.-S. (2022). Factors Related to Suicidal Ideation and Prediction of High-Risk Groups among Youngest-Old Adults in South Korea. Int. J. Environ. Res. Public Health.

[5] American Psychiatric Association (2022). Diagnostic and Statistical Manual of Mental Disorders, Fifth Edition, Text Revision (DSM-5-TR).

[6] Posner K., Brown G.K., Stanley B., Brent D.A., Yershova K.V., Oquendo M.A., Currier G.W., Melvin G.A., Greenhill L., Shen S. (2011). The Columbia–Suicide Severity Rating Scale: Initial validity and internal consistency findings from three multisite studies with adolescents and adults. Am. J. Psychiatry.

[7] Jeong J.-Y. (2020). Gender difference in socioeconomic factors affecting suicidal ideation and suicidal attempts among community-dwelling elderly: Based on the Korea Community Health Survey. Epidemiol. Health.

[8] Kim M., Lee Y.-H. (2018). Gender-specific factors related to suicidal ideation among community-dwelling stroke survivors: The 2013 Korean Community Health Survey. PLoS ONE.

[9] Jang J., Jung H.-S., Wang J., Kim S. (2021). Effects of health-related quality of life on suicidal ideation and depression among older Korean adults: A cross-sectional study. Psychiatry Investig..

[10] Goldney R.D., Fisher L.J., Wilson D.H., Cheok F. (2001). Suicidal ideation and health-related quality of life in the community. Med. J. Aust..

[11] Kim J.-H., Kwon J.-W. (2012). The impact of health-related quality of life on suicidal ideation and suicide attempts among Korean older adults. J. Gerontol. Nurs..

[12] Jo A., Nam S.-H. (2026). Health-risk behaviors and suicidal behavior in older adults: Structural equation modeling using a decade of Korean nationally representative data. Arch. Gerontol. Geriatr..

[13] Seo Y., Kim H.-Y., Choi K., Song S., Kim J. (2025). Contribution of chronic disease in predicting depression and suicidal ideation among the older adult population. Psychiatry Investig..

[14] Saito M., Watanabe R., Tamada Y., Takeuchi K., Tani Y., Kondo K., Ojima T. (2024). Social disconnection and suicide mortality among Japanese older adults: A seven-year follow-up study. Soc. Sci. Med..

[15] Torres Z., Martínez-Gregorio S., Fernández I., Tomás J.M., Oliver A. (2024). Suicidal ideation, social participation, loneliness, and mobility limitations: Longitudinal evidence in older European adults. Psicothema.

[16] Ki M., Lapierre S., Gim B., Hwang M., Kang M., Dargis L., Jung M., Koh E.J., Mishara B. (2024). A systematic review of psychosocial protective factors against suicide and suicidality among older adults. Int. Psychogeriatr..

[17] Jun H.J., Park H.-R. (2023). Factors associated with suicidal ideation among older adults in the community using data from the 2021 Community Health Survey. J. Korean Acad. Psychiatr. Ment. Health Nurs..

[18] Simon G.E., Rutter C.M., Peterson D., Oliver M., Whiteside U., Operskalski B., Ludman E.J. (2013). Does response on the PHQ-9 depression questionnaire predict subsequent suicide attempt or suicide death?. Psychiatr. Serv..

[19] Kroenke K., Strine T.W., Spitzer R.L., Williams J.B.W., Berry J.T., Mokdad A.H. (2009). The PHQ-8 as a measure of current depression in the general population. J. Affect. Disord..

[20] Korea Disease Control and Prevention Agency (2023). 2022 Korea Community Health Survey Raw Data User Guide and Reference Materials.

[21] Kroenke K., Spitzer R.L., Williams J.B. (2001). The PHQ-9: Validity of a brief depression severity measure. J. Gen. Intern. Med..

[22] Nam H.S., Kim K.Y., Kwon S.S., Koh K.W., Kind P. (2007). EQ-5D Korean Valuation Study Using Time Trade-Off Method.

[23] Park J.-I., Yang J.-C., Han C., Park T.W., Chung S.-K. (2016). Suicidal ideation among Korean elderly: Risk factors and population attributable fractions. Psychiatry.

[24] Pullenayegum E.M., Tarride J.-E., Xie F., Goeree R., Gerstein H.C., O’Reilly D. (2010). Analysis of health utility data when some subjects attain the upper bound of 1: Are Tobit and CLAD models appropriate?. Value Health.

[25] Graubard B.I., Korn E.L. (1999). Predictive margins with survey data. Biometrics.

[26] Benjamini Y., Hochberg Y. (1995). Controlling the false discovery rate: A practical and powerful approach to multiple testing. J. R. Stat. Soc. Ser. B.

[27] Choi M., Sempungu J.K., Kim M.-H., Han J., Lee Y.H. (2024). Happiness and its association with suicide ideation and attempt in Korea: The roles of socio-environmental, psychological, and health-related factors. J. Korean Med. Sci..

[28] Schuster N.A., Twisk J.W.R., ter Riet G., Heymans M.W., Rijnhart J.J.M. (2021). Noncollapsibility and its role in quantifying confounding bias in logistic regression. BMC Med. Res. Methodol..

[29] MacKinnon D.P., Krull J.L., Lockwood C.M. (2000). Equivalence of the mediation, confounding and suppression effect. Prev. Sci..

