# Supplementary material for: Associations Between Mental-Health Factors, Quality of Life, and Suicidal Ideation in Korean Elderly
Source: Healthcare (Basel). 2026 Sep 20;14(18):3109. doi: 10.3390/healthcare14183109 (PMC13606166; doi:10.3390/healthcare14183109)
Supplement: Supplementary file 1 [file healthcare-14-03109-s001.zip › healthcare-4550745-supplementary.pdf]

Supplementary materials

Associations Between Mental Health Factors, Quality of Life, and Suicidal Ideation in Korean Elderly
Manuscript healthcare-4550745

These tables provide missing-data summaries, selected sensitivity results, and complete coefficient estimates for three specified alternative models. All estimates account for the KCHS survey design. The outcome item asks about past-year thoughts of wanting to die and may include passive death wishes.

Table S1. Missingness and analysis-domain inclusion

Characteristic	Missing n	Required: logistic	Required: EQ-5D
**A. Variable-specific missingness among 79,434 respondents with a valid outcome**			
PHQ-8 total	325	Yes	Yes
PHQ-9 total (common-cohort criterion)	328	Yes	Yes
Sex	0	Yes	Yes
Age	0	Yes	Yes
Education	26	Yes	Yes
Spousal cohabitation	10	Yes	Yes
Basic livelihood security recipient	13	Yes	Yes
Economically active	2	Yes	Yes
Diagnosed hypertension	18	Yes	Yes
Diagnosed diabetes	7	Yes	Yes
Self-rated health	1	Yes	No
Happiness	246	Yes	No
Perceived stress	46	Yes	No
EQ-5D index	17	Yes	Yes

B. Included and excluded respondents in each analysis domain

Characteristic	Logistic: included n = 78,838	Logistic: excluded n = 596	EQ-5D: included n = 79,033	EQ-5D: excluded n = 401
Age, years	73.8439 (0.0349)	79.2552 (0.5625)	73.8532 (0.0349)	78.9441 (0.7798)
Women, proportion	0.5443 (0.0018)	0.6553 (0.0304)	0.5446 (0.0018)	0.6087 (0.0399)
PHQ-8 total	2.7441 (0.0178)	4.2601 (0.3029)	2.7473 (0.0178)	3.3155 (0.5079)
EQ-5D index	0.8760 (0.0008)	0.7365 (0.0165)	0.8757 (0.0008)	0.7527 (0.0203)
Outcome positive, proportion	0.0983 (0.0015)	0.2386 (0.0289)	0.0986 (0.0015)	0.2291 (0.0361)

Panel A uses outcome-valid respondents as the denominator; missing counts can overlap. Both domains required valid PHQ-8 and PHQ-9 totals so that PHQ replacement analyses used identical respondents. Panel B reports survey-weighted means or proportions (SE); group sizes are unweighted. Logistic refers to main Tables 2 and 3, and EQ-5D to main Table 4. The comparisons describe potential selection differences and do not establish that missingness is ignorable.

Table S2. Sensitivity analyses and additional standardized prevalences

The table reports estimates relevant to model specification, expanded adjustment, and the interpretation of the main findings. Rows have different estimands; aORs per score point are not directly comparable to category contrasts. Each analysis uses the stated common domain.

Analysis and quantity	Estimate (95% CI), unless stated otherwise	*p or q*	n
**A. Alternative specifications and functional form**			
PHQ-8, continuous sensitivity aOR per point	1.175 (1.161 to 1.188)	p < 0.001	78,838
PHQ-9 replacement aOR per point	1.203 (1.189 to 1.217)	p < 0.001	78,838
PHQ-8: 5–9 vs 0–4 Five-band R sensitivity	2.612 (2.383 to 2.862)	p < 0.001	78,838
PHQ-8: 10–14 vs 0–4 Five-band R sensitivity	4.146 (3.577 to 4.805)	p < 0.001	78,838
PHQ-8: 15–19 vs 0–4 Five-band R sensitivity	5.684 (4.509 to 7.165)	p < 0.001	78,838
PHQ-8: 20–24 vs 0–4 Five-band R sensitivity	8.268 (5.221 to 13.091)	p < 0.001	78,838
PHQ-8 squared (log-odds B)	−0.0100 (−0.0114 to −0.0087)	p < 0.001	78,838
Age squared (log-odds B)	0.000109 (−0.000571 to 0.000789)	p = 0.753	78,838
Happiness squared (log-odds B)	0.0090 (0.0012 to 0.0169)	p = 0.023	78,838
**B. Expanded covariates: paired E0 and E1 estimates**			
PHQ-8: 5–9 vs 0–4	E0: 2.582 (2.356 to 2.830)<br>E1: 2.536 (2.313 to 2.781)	E0 p < 0.001<br>E1 p < 0.001	77,892
PHQ-8: 10–14 vs 0–4	E0: 4.103 (3.539 to 4.756)<br>E1: 3.947 (3.404 to 4.577)	E0 p < 0.001<br>E1 p < 0.001	77,892
PHQ-8: 15–24 vs 0–4	E0: 5.913 (4.770 to 7.329)<br>E1: 5.670 (4.559 to 7.052)	E0 p < 0.001<br>E1 p < 0.001	77,892
Happiness, per point	E0: 0.771 (0.752 to 0.790)<br>E1: 0.778 (0.760 to 0.798)	E0 p < 0.001<br>E1 p < 0.001	77,892
Stress: a little vs hardly any	E0: 1.453 (1.313 to 1.608)<br>E1: 1.471 (1.329 to 1.627)	E0 p < 0.001<br>E1 p < 0.001	77,892
Stress: much vs hardly any	E0: 2.470 (2.188 to 2.787)<br>E1: 2.486 (2.203 to 2.806)	E0 p < 0.001<br>E1 p < 0.001	77,892
Stress: very much vs hardly any	E0: 2.642 (2.142 to 3.260)<br>E1: 2.684 (2.176 to 3.311)	E0 p < 0.001<br>E1 p < 0.001	77,892
Anxiety/depression: some vs none	E0: 2.252 (2.047 to 2.477)<br>E1: 2.232 (2.029 to 2.456)	E0 p < 0.001<br>E1 p < 0.001	77,892
Anxiety/depression: severe vs none	E0: 4.109 (3.220 to 5.245)<br>E1: 4.029 (3.156 to 5.143)	E0 p < 0.001<br>E1 p < 0.001	77,892
**C. Usual activities and single-PSU variance sensitivity**			
Usual activities: some problems	UA-A: 3.358 (3.109 to 3.626)<br>UA-B: 1.405 (1.279 to 1.543)<br>UA-C: 1.151 (1.022 to 1.297)<br>UA-D: 1.085 (0.961 to 1.225)	NA	78,838

Analysis and quantity	Estimate (95% CI), unless stated otherwise	*p or q*	n
Usual activities: severe problems	UA-A: 6.141 (5.321 to 7.088)<br>UA-B: 1.080 (0.900 to 1.296)<br>UA-C: 0.776 (0.592 to 1.019)<br>UA-D: 0.695 (0.528 to 0.914)	NA	78,838
Pain/discomfort: some problems	Conservative aOR: 1.130 (1.010 to 1.265)	Primary q = 0.042<br>Conservative q = 0.057	78,838
Pain/discomfort: severe problems	Conservative aOR: 1.211 (1.011 to 1.450)	Primary q = 0.046<br>Conservative q = 0.063	78,838
**D. EQ-5D secondary-analysis sensitivities**			
Outcome-status coefficient PHQ-9 replacement	B −0.0432 (−0.0490 to −0.0374)	p < 0.001	79,033
Two-part standardized means and combined contrast	Outcome no: 0.8806<br>Outcome yes: 0.8301<br>Yes minus no: −0.0506 (point estimate)	Not estimated	79,033
Outcome status × PHQ-8	B −0.0003 (−0.0018 to 0.0012)	p = 0.703	79,033
Outcome status × PHQ-9	B 0.0008 (−0.0006 to 0.0021)	p = 0.258	79,033
**E. Additional model-standardized prevalences**			
Stress: hardly any	7.0% (6.5 to 7.5)	NA	78,838
Stress: a little	9.2% (8.9 to 9.6)	NA	78,838
Stress: much	13.3% (12.6 to 14.0)	NA	78,838
Stress: very much	13.8% (12.2 to 15.6)	NA	78,838
Anxiety/depression: no problems	7.5% (7.1 to 7.8)	NA	78,838
Anxiety/depression: some problems	13.5% (12.9 to 14.2)	NA	78,838
Anxiety/depression: severe problems	20.0% (17.2 to 23.0)	NA	78,838

aOR, adjusted odds ratio; B, regression coefficient; CI, confidence interval. B is on the log-odds scale in group A and the EQ-5D index scale in group D. The displayed p values are individual exploratory tests. The five-band PHQ-8 reference is 0–4. Quadratic terms were tested separately: PHQ-8 in a continuous specification, and age and happiness in the categorical primary specification.

E0 and E1 use the same 77,892 respondents. E0 replaces spousal cohabitation with marital-status indicators; E1 adds the further covariates specified in Section 2.3.7 while retaining the other primary terms. UA-A includes demographic/clinical covariates and usual activities; UA-B adds categorical mental-health measures; UA-C adds the remaining physical EQ-5D dimensions; UA-D adds anxiety/depression. UA-C and UA-D correspond to M4 and M5.

The conservative analysis centers single-PSU contributions at the grand mean; both q columns use the original 30-term BH family. The two-part contrast combines predictions for each of 79,033 respondents before averaging; its non-ceiling component was fitted in 47,084. The combined contrast has no joint CI or p value. NA means no test is reported. Nonsignificant interactions do not establish equivalence. Group E uses survey-weighted standardization with logit-transformed Taylor CIs (df = 14,181); PHQ-8 estimates appear in Figure 2.

Table S3. Coefficients of the specified alternative models

All fitted terms, including intercepts, are reported for the three models below. The first model predicts the suicidal-ideation item, the second predicts full health, and the third estimates the EQ-5D index below 1. These columns have different outcomes and are not a sequential adjustment comparison.

Parameter	EQ-5D index replacement aOR (95% CI)	*p*	Full health aOR (95% CI)	*p*	Index below 1 B (95% CI)	*p*
Intercept	0.878 (0.481 to 1.600)	0.670	119.809 (88.041 to 163.041)	<0.001	1.2352 (1.2101 to 1.2602)	<0.001
Suicidal-ideation item: yes vs no	NA	NA	0.423 (0.378 to 0.473)	<0.001	−0.0362 (−0.0423 to −0.0301)	<0.001
PHQ-8, per point	1.200 (1.187 to 1.214)	<0.001	0.744 (0.735 to 0.753)	<0.001	−0.0139 (−0.0146 to −0.0132)	<0.001
Women (ref: men)	0.950 (0.874 to 1.032)	0.225	0.564 (0.539 to 0.591)	<0.001	0.0022 (−0.0016 to 0.0061)	0.258
Age, per year	0.990 (0.984 to 0.996)	0.001	0.943 (0.939 to 0.946)	<0.001	−0.0054 (−0.0057 to −0.0051)	<0.001
Middle/high school	0.868 (0.797 to 0.945)	0.001	1.590 (1.514 to 1.671)	<0.001	0.0211 (0.0172 to 0.0249)	<0.001
College or higher	0.764 (0.645 to 0.905)	0.002	1.986 (1.846 to 2.137)	<0.001	0.0381 (0.0316 to 0.0446)	<0.001
Living with spouse	0.707 (0.649 to 0.771)	<0.001	1.092 (1.038 to 1.150)	<0.001	−0.0009 (−0.0047 to 0.0029)	0.640
Basic livelihood security recipient	1.104 (0.981 to 1.243)	0.100	0.665 (0.604 to 0.732)	<0.001	−0.0247 (−0.0311 to −0.0182)	<0.001
Economically active	0.902 (0.824 to 0.987)	0.024	1.314 (1.254 to 1.377)	<0.001	0.0328 (0.0298 to 0.0357)	<0.001
Diagnosed hypertension	1.007 (0.930 to 1.090)	0.864	0.799 (0.764 to 0.836)	<0.001	−0.0112 (−0.0146 to −0.0079)	<0.001
Diagnosed diabetes	1.097 (1.005 to 1.197)	0.038	0.929 (0.882 to 0.978)	0.005	−0.0114 (−0.0154 to −0.0074)	<0.001
Self-rated health, per level	0.892 (0.846 to 0.940)	<0.001	NA	NA	NA	NA
Happiness, per point	0.756 (0.737 to 0.774)	<0.001	NA	NA	NA	NA
Stress, per level	1.612 (1.528 to 1.701)	<0.001	NA	NA	NA	NA
EQ-5D index	0.390 (0.308 to 0.493)	<0.001	NA	NA	NA	NA

EQ-5D index replacement: n = 78,838; the logistic sensitivity uses continuous PHQ-8, self-rated health, happiness, stress, demographic/clinical covariates, and the EQ-5D index instead of its five dimensions. Full health: n = 79,033; logistic model for EQ-5D = 1. Index below 1: n = 47,084; linear model restricted to non-ceiling respondents. The two-part components include outcome status, PHQ-8, sex, age, education, spousal cohabitation, basic livelihood security, economic activity, hypertension, and diabetes. Both components are then predicted for the full 79,033-person target. Education uses elementary school or below as the reference. Binary indicators compare yes with no; NA means that the predictor is not included in that model. CIs and p values are design-based, without an additional table-wide multiplicity adjustment.
